# Acknowledgement of manuscript reviewers 2015

**DOI:** 10.1186/s13010-016-0034-4

**Published:** 2016-03-01

**Authors:** Kevin G. Donovan, James Giordano

**Affiliations:** Georgetown University Medical Center, Washington, USA

## Abstract

The editors of *Philosophy, Ethics, and Humanities in Medicine* would like to thank all our reviewers who have contributed to the journal in Volume 10 (2015).

**A Pascalev**

Bulgaria

**Roland Benedikter**

Italy

**John Shook**

USA

**Silke Schicktanz**

Germany

**Dan Stein**

South Africa

**Patrick Brown**

UK

**David Miller**

USA

**Michael Schwartz**

USA

**Ray Greek**

USA

**Jeff Collmann**

USA

**Elizabeth Davenport**

USA

**Guillermo Palchik**

USA

**Joan Engebretson**

USA

